# Supplementary material for: Identification of photoperiod-induced specific miRNAs in the adrenal glands of Sunite sheep (Ovis aries)
Source: Front Vet Sci. 2022 Jul 22;9:888207. doi: 10.3389/fvets.2022.888207 (PMC9354845; doi:10.3389/fvets.2022.888207)
Supplement: Supplementary file 1 [file Data_Sheet_1.ZIP › Supplementary file/miRDeep2 file.zip!/miRDeep2 file/Know_Structure/SP42A1/oar-mir-150.pdf]

miRBase precursor : oar-mir-150
Total read count : 600
oar-miR-150 read count : 600
remaining reads : 0

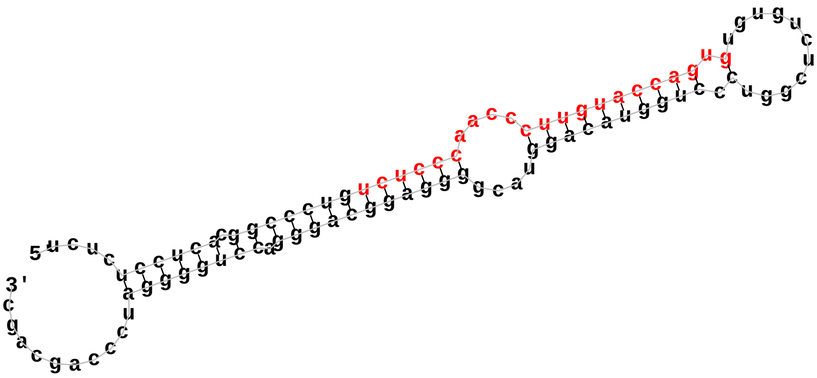

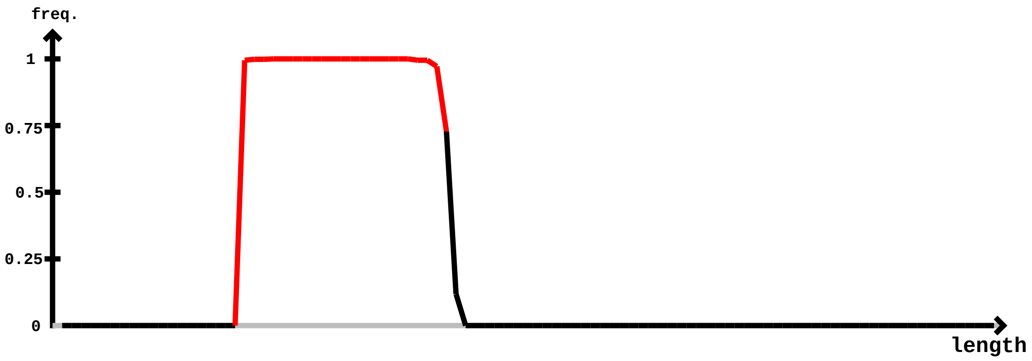


```
                  oar-miR-150
5'- ucucuccucacggcccugucucccaacccuuguaccagugugugucucggucccugguacagguacggggaggcagggaccuggggaucccagcagc   -3'    exp
    ....((((((.(((((((((((((....((((((((((.(...........).))))))))))....))))))))))).))))))))..........   reads    mm    sample
    ..................ucucccaacccuuguacc.............................................................   3        0     seq
    ..................ucucccaacccuuguaccag...........................................................   14       0     seq
    ..................ucucccaacccuuguaccagu..........................................................   145      0     seq
    ..................ucucccaacccuuguaccagug.........................................................   365      0     seq
    ..................ucucccaacccuuguaccagugu........................................................   70       0     seq
    ...................cucccaacccuuguaccagu..........................................................   1        0     seq
    ...................cucccaacccuuguaccagugu........................................................   1        0     seq
    .....................cccaacccuuguaccagug.........................................................   1        0     seq
```
